# A national geostatistical survey for schistosomiasis and soil-transmitted helminths in Zimbabwe following interruption of preventive chemotherapy

**DOI:** 10.1186/s44263-026-00301-x

**Published:** 2026-09-22

**Authors:** Nicholas Midzi, Masceline J. Mutsaka-Makuvaza, Isaac Phiri, Rebecca Mwabvu, Kennedy Mubaiwa, Elyse Greenblatt, Alison Ower, Karen Palacio, Neerav Dhanani, Claudio Fronterre, Fiona M Fleming, Rachel L Pullan, Joseph WS Timothy

**Affiliations:** 1 National Institute of Health Research, Ministry of Health and Child Care, Harare, Zimbabwe; 2 Department of Microbiology and Parasitology, University of Rwanda, Butare, Rwanda; 3 Department of Epidemiology and Disease Control, Ministry of Health and Child Care, Harare, Zimbabwe; 4 Higherlife Foundation, Harare, Zimbabwe; 5 The END Fund, New York, United States of America; 6 Unlimit Health, London, UK; 7 Centre for Health Informatics, Computing and Statistics, Lancaster University, Lancaster, UK; 8 Department of Disease Control, London School of Hygiene and Tropical Medicine, London, UK

**Keywords:** Infectious disease epidemiology, Schistosomiasis, Soil-transmitted helminthiasis, Neglected tropical diseases, Zimbabwe, Elimination as a public health problem, Precision public health, Precision mapping

## Abstract

**Background:**

Schistosomiasis and soil-transmitted helminthiases (STH) are targeted for elimination as public health problems (EPHP) in Zimbabwe by 2030. A 2018 impact survey demonstrated prevalence below EPHP thresholds following seven rounds of annual preventive chemotherapy (PC). However, the COVID-19 pandemic caused a three-year suspension of PC. We conducted a nationwide geostatistical impact assessment to estimate prevalence at ward-level and evaluated impact of PC interruption relative to 2018 results using a quasi-experimental design.

**Methods:**

The cross-sectional survey in 2021 targeted 11,600 school-aged children (SAC) and sub-sets of pre-SAC and adults. Sampling was optimised to achieve 90% correct classification of wards (*N* = 1,961) using geostatistical predictions from 2018 impact data. 200 schools were selected with inclusion proportional to predicted *Schistosoma haematobium* prevalence. All SAC provided urine and stool samples analysed by urine filtration and Kato-Katz. Geostatistical models incorporating environmental covariates were fitted and used to generate high-resolution prevalence predictions.

**Results:**

Data from 11,190 participants indicated a national schistosomiasis prevalence of 7.0% (95% CI 5.0-9.8%) including focal recrudescence of *S. mansoni.* Heavy-intensity infection recrudesced above 1% EPHP threshold (1.4%, 95% CI 0.9–2.0). STH prevalence increased though remained low (1.4%, 95% CI 1.0-2.1%), with few moderate-heavy infections (< 0.1%, 95% CI < 0.1–1.2). Geostatistical predictions delineated key spatial heterogeneity: 98.9% of wards exceeded 2% schistosomiasis prevalence (17,909,432 people), 37.2% required annual PC (≥ 10% prevalence; 6,473,455 people), and 33.4% wards exceeded 2% prevalence for STH (biennial PC).

**Conclusions:**

PC suspension led to recrudescence of schistosomiasis and smaller increases in STH prevalence. These findings emphasise the risk of pausing PC even in low or moderate prevalence settings. Continued surveillance and tailored PC are essential to achieve 2030 goals, with geostatistical outputs supporting precision interventions.

**Supplementary Information:**

The online version contains supplementary material available at https://doi.org/10.1186/s44263-026-00301-x.

## Background

Schistosomiasis and soil-transmitted helminthiases (STH) are neglected tropical diseases (NTDs) caused by parasitic worm infections, transmitted through contact with contaminated water and soil respectively [1, 2]. The burden of these diseases is particularly high in sub-Saharan Africa. The World Health Organization (WHO) estimated that in 2023, 231 million people across 41 countries required preventive chemotherapy (PC) for schistosomiasis and 233 million people across 38 countries required PC for STH [3]. Over the past 20 years, large-scale PC using praziquantel for schistosomiasis and benzimidazoles for STH has been rolled out across endemic countries to reduce prevalence and morbidity [4]. Building on these foundations, the WHO 2030 NTD Roadmap now targets both diseases for elimination as a public health problem (EPHP) by 2030 [5]. EPHP is defined as low prevalence of heavy (< 1% schistosomiasis) or moderate to heavy intensity infections (< 2% STH). Recent guidance has also recommended transitioning schistosomiasis control from district- to community-level PC implementation [6]. As a result, NTD programmes must now generate granular prevalence estimates to inform future PC strategies – prompting a shift in implementation units (IU) from district to sub-districts.

Schistosomiasis and STH are endemic in Zimbabwe, with early studies documenting their widespread distribution across diverse ecologies [7, 8]. A national baseline survey by the Ministry of Health and Child Care (MoHCC) of Zimbabwe in 2010 reported schistosomiasis prevalence at 22.7% and STH (predominantly hookworm and *Ascaris lumbricoides*) at 5.5% among school-aged children. *Schistosoma haematobium* showed broader geographic distribution, whilst *S. mansoni* was more geographically restricted, with foci of high prevalence seen in the northern and eastern region of the country [9]. In response, the MoHCC implemented seven rounds of integrated PC from 2012 to 2018, targeting school-aged children (SAC). A 2018 impact assessment demonstrated EPHP for schistosomiasis (< 1% heavy intensity infection) and STH (< 2% moderate or heavy intensity) in SAC [10]. Although a conventional district-level survey, subsequent model-based geostatistical analysis of the impact assessment data confirmed consistent STH prevalence reductions and suggested cessation of PC in 54 districts, with reduced frequency in the remaining six.

From 2018, schistosomiasis and STH control activities in Zimbabwe were disrupted for three years due to the Sars-Cov-2 pandemic and subsequent vaccination campaigns. Atypically high reports of STH and schistosomiasis-related clinical complications from district-level health surveillance systems resulted in reactive PC in seven out of 91 districts in 2020. The districts receiving PC were in Manicaland (*n* = 1), Mashonaland Central (*n* = 3), Mashonaland West (*n* = 1) and Midlands (*n* = 2) provinces with mean coverage of 77.2% for praziquantel and 70.5% for albendazole. These findings raised concerns about possible recrudescence of infection and subsequent changes to the classification of implementation units ahead of the planned reinitiation of PC in 2022. In response, the MoHCC prioritised a second national impact assessment survey, aligned with updated WHO guidance emphasising epidemiological data at finer spatial scales, specifically sub-district (ward) level. Building on the 2018 geostatistical analysis [10], the 2021 national impact assessment adopted a model-based geostatistical (MBG) framework for both design and analysis. By leveraging spatial autocorrelation and associations with environmental covariates, this approach improves precision and allows reliable prediction in unsampled areas [11]. The MBG approach also integrated prior data and uncertainty to optimise design and inference [12]. Here, we present results from Zimbabwe’s 2021 impact assessment to update ward-level prevalence estimates to guide national decisions for schistosomiasis and STH.

## Methods

Reporting of this study has been verified in accordance with the Strengthening the Reporting of Observational Studies in Epidemiology (STROBE) checklist (Supplementary Material 1) [13].

### Study design and participants

A population-based cross-sectional survey was conducted between November and December 2021 targeting 11,600 school-aged children (ages 9–14 years) nationwide. We employed a geostatistical design and analysis framework in which schools were the primary sampling units (PSUs). Our primary aim was to correctly classify 90% of wards (sub-districts) into prevalence categories defined by the MoHCC and WHO for infection with any schistosomiasis species i) < 2%, ii) 2 to < 10%, iii) 10 to < 50% and iv) ≥ 50%, and any STH species i) < 2%, ii) 2 to < 5%, iii) 5 to < 10% and iv) ≥ 10%. We compared findings to those of a published 2018 nationwide impact survey as a quasi-experimental before-after study to quantify the impact of PC interruption.

We also used results from the 2018 schistosomiasis and STH survey [10] to inform the design and analysis of the 2021 impact assessment by maximizing efficiency when determining the sample size. First, we generated a geostatistical predictive surface of *S. haematobium* across Zimbabwe using 2018 impact assessment data (Supplementary Material 2, Table S1-2, Figure S1). We then simulated potential survey designs across predicted realizations - varying sampling designs, analysis approaches, sites, and PSUs sampled – to specify a design which correctly classified 90% of wards with minimal survey effort (Supplementary Material 2, Figure S2-3). The optimal design targeted 200 PSUs using a spatially-regulated design with selection proportional to exceedance probability of 2% prevalence for *S. haematobium*. Within PSUs, 58 children aged 9–14 and stratified by sex were selected per school. In a random sub-sample of locations (*n* = 25), pre-SAC and adults were sampled alongside SAC. Results and methods from the 2018 impact assessment are included in the supplementary information and as previously reported by Midzi and colleagues [10].

### Study procedures

All study participants were asked to provide their name, age, sex, school grade and responses to a short set of behaviour-exposure questions, plus a sample of mid-morning urine and fresh stool. Urine and stool samples were examined on the day of collection. *Schistosoma haematobium* infection was assessed by urine filtration, with intensity of infection expressed as eggs per 10ml. *Schistosoma mansoni* and STH were assessed by duplicate Kato-Katz thick smears and intensity expressed as eggs per gram of feces. A 10% subset of slides were re-read by blinded senior technicians for quality control and any discrepancies resolved on-site. Results were recorded on pre-designed paper-based forms during data collection and double-entered into SPSS.

### Statistical analyses

Data were managed and analysed in R (version 4.3.2, R Core Team 2023). All data and analysis code are available within Supplementary Materials 3–8. Primary outcomes were categorized as prevalence of infection and heavy intensity infection (*S. haematobium* ≥ 50 eggs per 10 ml of urine; *S. mansoni* ≥ 400 eggs per gram of stool), or moderate and heavy intensity infection (*A. lumbricoides* ≥ 5,000, *hookworm* ≥ 1,000, *T. trichiura* ≥ 2,000 eggs per gram of stool). We calculated national and provincial prevalence estimates using design-based inference under a one-stage cluster design with PSUs weighted by the reciprocal of inclusion probability. We estimated robust confidence intervals using Wald’s method (survey package, version 4.4.2) and we report findings as survey-adjusted prevalence values at national and province-level, with associated 95% confidence intervals.

School-level prevalence data were then analysed using a geostatistical modelling framework, an extension of mixed-effects logistic regression that incorporates spatial autocorrelation between PSUs and supports interpolation of prevalence into unsampled areas. For each species, we fitted a geostatistical model as follows:

$$\:log\left\{\frac{p\left({x}_{i}\right)}{1-\:p\left({x}_{i}\right)}\right\}=\:\alpha\:+{d{\left({x}_{i}\right)}^{{\prime\:}}\beta\:+S\left({x}_{i}\right)+Z\left({x}_{i}\right)}_{}$$

Where $\:{x}_{i}$ is the sampling location,$\:\:S\left({x}_{i}\right)$is a zero mean Gaussian process with an exponential correlation function, $\:d{\left({x}_{i}\right)}^{{\prime\:}}\beta\:$ is a vector of covariates at each location and $\:Z\left({x}_{i}\right)$ is a random effect that accounts for spatially unstructured residual variance. Models were fit using Monte Carlo maximum likelihood (MCML) (R, PrevMap v1.5.4) and validated by comparing semivariograms of residuals from the observed data (with the spatial random effect set to zero) to semivariograms derived from 1,000 simulations from the full fitted mode.

For each species, we first fitted a model without covariates. However, for each schistosomiasis species, inclusion probability was included as a fixed effect to account for the sampling design. We then added covariates selected from a suite of 30 across five risk domains (Supplementary Material 2, Table S3). Domains were defined based on the requirement that each provides an independent contribution to risk of schistosomiasis infection at the scales measured in the data. We did not limit the number of covariates selected per domain. All covariates were screened for collinearity and removed if pairwise Pearson correlation was ≥ 0.8, then added in a stepwise fashion in a non-spatial framework. Each mode at this stage was evaluated for improvement in model fit to determine inclusion of a covariate in a final geostatistical model. For STH, prevalence of species other than *A. lumbricoides* was low (0.2% or below) so we fit a single model to infection with any STH species. We did not fit geostatistical models to intensity data due to limited observations with outcomes.

To predict prevalence, we generated 1,000 samples from the predictive distribution at each 1 km² grid cell, using MCML parameters. Ward-level prevalence and exceedance probabilities were derived from the joint pixel distribution, targeting population-weighted prevalence:

$$\:T=\:\int\:pd\left(x\right)p\left(x\right)$$

Where $\:pd\left(x\right)$ is the population density derived from a 1 km gridded population surface; WorldPop unconstrained and UN-adjusted estimates [14] from most recently available year (2020). Wards were classified into prevalence categories (e.g. >=2 to < 10%) using a conservative rule: a ward was assigned to a category when the probability of exceeding its lower threshold was at least 30% and the probability of exceeding its upper threshold was below 30%. This strategy prioritised minimising the risk of ‘under-treatment’ by only recommending a lower intervention category when there was at least 70% certainty that prevalence fell below that threshold. We estimated and report the population living within each ward and prevalence category by extracting 1 km WorldPop raster data using the ward shapefile.

## Results

### Sample sites and populations

Complete datasets were available from 9,778 SAC in 177 schools across eight provinces (two urban provinces – Bulawayo and Harare – had no schools selected during sampling; Supplementary Material 2, Figure S4). In addition, data from 725 PSAC and 750 adults were also collected from the sub-sample of 25 sites. The sample population was split evenly by sex (Table 1) and the mean age of the sample population was 12.3 years (SD 7.4). The median distance between PSUs was 19.8 km (IQR 13.7–30.2; Supplementary Material 2, Figure S4).

### Distribution of schistosomiasis

**Fig. 1:**
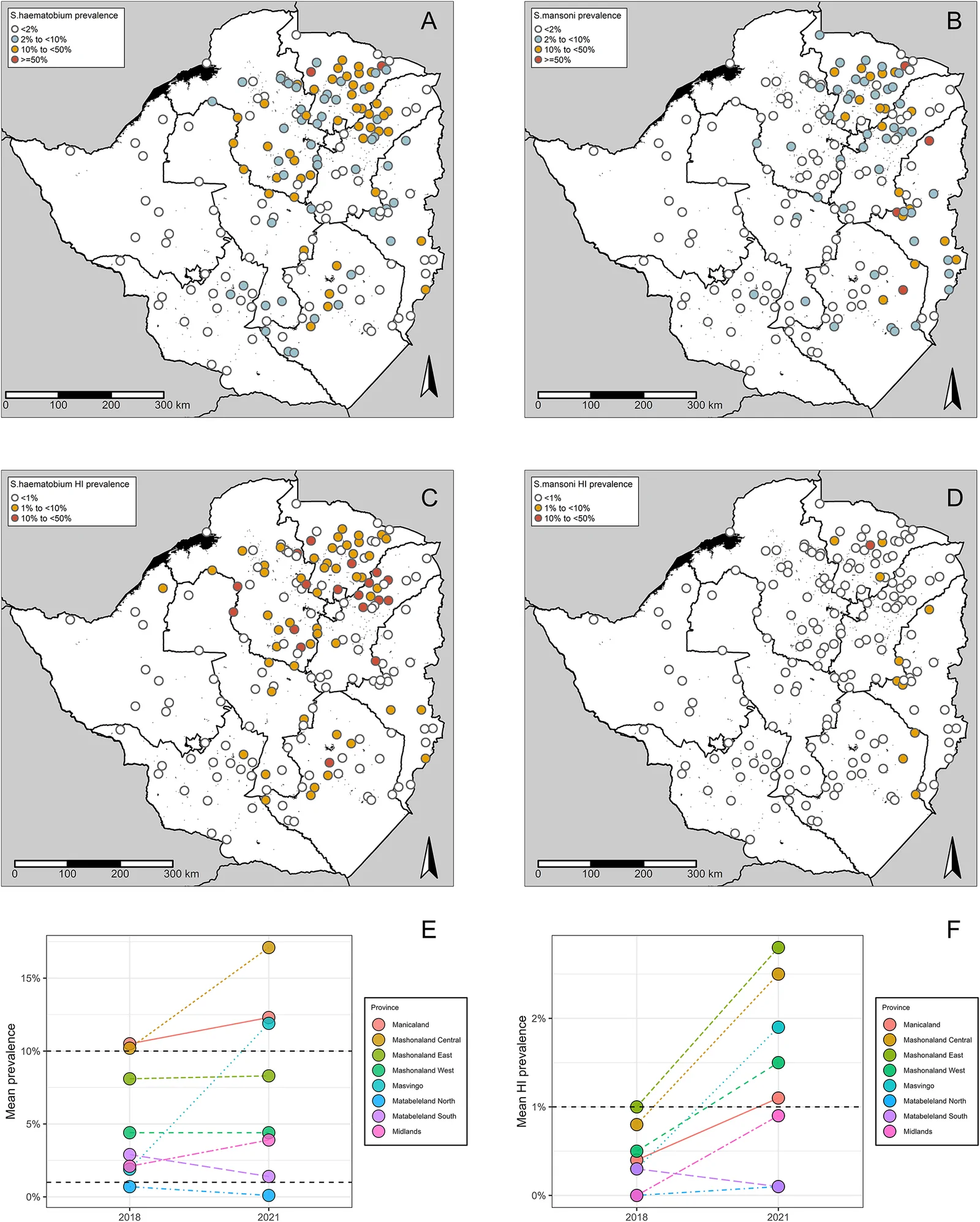
Site-level prevalence of infection with *Schistosoma haematobium* (**A**) or *S. mansoni* (**B**). Site-level prevalence of heavy intensity infection with *S. haematobium* (**C**) or *S. mansoni* (**D**). Changes in mean province infection prevalence with any schistosomiasis infection between 2018 and 2021 surveys (**E**). Changes in heavy intensity infection with any schistosomiasis species (**F**)

The estimated survey prevalence for any schistosomiasis infection was 7.0% (95% CI 5.0-9.8%; Table 1), 4.0% for *S. haematobium* (95% CI 2.9–5.6%) and 3.8% for *S. mansoni* (95% CI 2.2–6.5%) with 116 cases of combined species infection (0.5% 95% CI 0.3–0.9%). All outcomes were notably higher than estimates from the 2018 impact assessment yet remained marginally within the confidence bounds of their respective 2018 estimate (Supplementary Material 2, Table S1; any schistosomiasis infection 2018 estimate; 5.1% 95% CI 4.6–5.5). Most sites demonstrated infection with *S. haematobium* (62.7%) or, *S. mansoni* (51.4%) with greater occurrence of *S. haematobium* expected given the inclusion weights of selected PSUs. There were few sites with high prevalence (≥ 50%) for either *S. haematobium* (2, 1.1%) or *S. mansoni* (4, 2.3%; Fig. 1A-B). We observed a much greater proportion of moderate prevalence (≥ 10% and < 50%) locations for *S. haematobium* (45, 25.4%) than *S. mansoni* (18, 10.1%). Despite increasing sampling probability in areas of known schistosomiasis transmission, a high proportion of sites were < 2% (*S. haematobium;* 84, 47.5%; *S. mansoni*; 110, 62.1%) or between 2 and < 10% for both species (*S. haematobium;* 46, 26.0%; *S. mansoni*; 45, 25.4%). Design-adjusted estimates for all provinces included in the survey (Table 1) indicate modest yet consistent increases in prevalence of both species across endemic areas since the 2018 impact assessment survey (Supplementary Material 2 Table S1, Fig. 1E). There were striking increases in prevalence driven by *S. mansoni* in both Mashonaland Central and Masvingo, though with notable uncertainty. Whilst Mashonaland Central exhibited moderate prevalence in 2018 (≥ 10%), Masvingo was low (< 2%).

**Table 1:** Sample numbers and disaggregation by sex and age for each schistosomiasis outcome measure (*Schistosoma mansoni*,* Schistosoma haematobium*, any *Schistosoma* species infection)

	S. mansoni	Heavy intensity	S. haematobium	Heavy intensity	Any Schistosoma Spp.	Heavy intensity
Survey sites	177		177		177	
Examined (N)	10,676		11,190		11,269	
Infected (N)	550	124	848	248	1,282	274
Sex; Female N (%)	5,289 (49.5%)		5,547 (49.6%)		5,592 (49.6%)	
Age (Mean, SD)	12.2 (7.3)		12.3 (7.4)		12.3 (7.4)	
**National prevalence (95% CI)**						
Overall	3.8 (2.2–6.5)	0.2 (0.1–0.6)	4.0 (2.9–5.6)	1.2 (0.8–1.7)	7.0 (5.0–9.8)	1.4 (0.9–2.0)
**Province prevalence (95% CI)**						
Manicaland	8.1 (3.4–18.1)	3.0 (0.1–1.4)	5.8 (1.6– 18.9)	0.8 (0.2–2.5)	12.3 (5.3–26.0)	1.1 (0.4–2.7)
Mashonaland Central	9.5 (5.0–17.2)	0.4 (0.2–1.3)	11.7 (6.6–20.0)	2.2 (1.2–3.9)	17.1 (10.1–27.5)	2.5 (1.4–4.6)
Mashonaland East	3.2 (1.2–8.3)	0.1 (< 0.1– 0.5)	6.2 (3.0-12.2)	2.8 (1.1–6.9)	8.3 (4.0–16.6)	2.8 (1.1–7.1)
Mashonaland West	0.7 (0.3–1.3)	0 (< 0.1 – <0.1)	4.0 (1.5–10.3)	1.6 (0.5–4.3)	4.4 (1.9–10.0)	1.5 (0.5–4.3)
Masvingo	8.9 (3.2–22.4)	0.8 (0.3–2.7)	3.4 (1.5–7.8)	1.1 (0.4–3.5)	11.9 (5.7–23.3)	1.9 (0.9–4.1)
Matabeleland North	0 (0 - <0.1)	0 (< 0 – <0.1)	0.1 (< 0.1–0.7)	0.1 (< 0.1–0.7)	0.1 (< 0.1–0.7)	0.1 (< 0.1–0.7)
Matabeleland South	0.3 (0.1–1.1)	0 (0– <0.1)	1.1 (0.3–3.5)	0.1 (< 0.1–0.7)	1.4 (0.5–4.1)	0.1 (< 0.1–0.7)
Midlands	0.2 (< 0.1–0.9)	0 (0– <0.1)	3.7 (1.2–11.1)	0.9 (0.2–3.2)	3.9 (1.3–11.2)	0.9 (0.2–3.2)

Design-based estimates of prevalence at national and province level

**Fig. 2:**
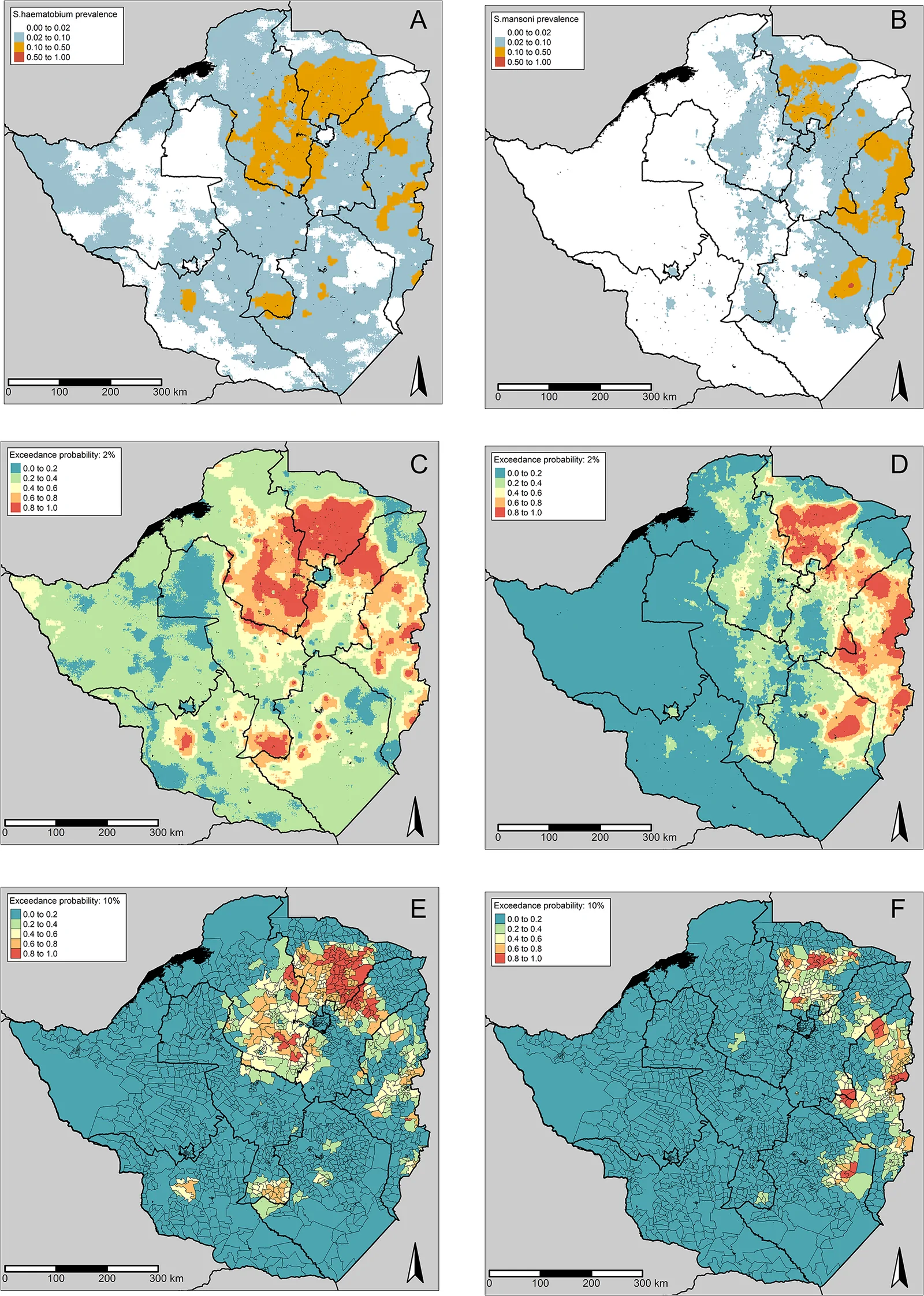
Predicted mean prevalence of infection with *Schistosoma haematobium* (**A**) or *S. mansoni* (**B**) using fitted geostatistical models. Estimated probability of exceeding 2% infection prevalence for *S. haematobium* (**C**) or *S. mansoni* (**D**). Population weighted probability of exceeding 2% prevalence at ward-level for *S. haematobium* (**E**) or *S. mansoni* (**F**)

The crude prevalence of heavy intensity infection with any *Schistosoma spp.* infection was 2.4% (95% CI 1.84–3.02%) across the total survey population. Adjusted for survey design, the estimated prevalence remained above the 1% EPHP threshold (1.4% 95% CI 0.9-2.0%; Table 1). Importantly this indicates that Zimbabwe has moved from below the schistosomiasis EPHP threshold in 2018 (0.4%, 95% CI 0.3–0.6) to exceeding it in 2021. Most heavy intensity infections were caused by *S. haematobium* (1.2% 95% CI 0.8–1.7%) and concentrated in the north of Zimbabwe (Fig. 1C-D). The remaining prevalence attributable to *S. mansoni* heavy intensity infections was low (0.2% 95% CI 0.1–0.6%), though no heavy intensity infections were identified in 2018. Province-level estimates (Table 1) highlight the concentration of high burden infections within six provinces, with similar distribution to 2018 (Supplementary Material 2, Table S1) though at higher prevalence and most above the 1% EPHP threshold (Fig. 1F). Geostatistical models were fitted to each species, with both exhibiting short-range spatial autocorrelation (estimated range < 20 km; Supplementary Material 2, Table S4 and Figure S5-6). For *S. haematobium*, predictive performance was not improved by environmental covariates, although survey inclusion probability was highly informative. In contrast, model fits for *S. mansoni* were improved by inclusion of precipitation, temperature, land cover, socioeconomic and healthcare access covariates (Supplementary Material 2,Table S5). Figure 2A-B shows the mean predicted prevalence and probability of exceeding 2% for each species. *Schistosoma haematobium* displayed a broader geographic range, with many areas predicted between 2 and 10%. However, substantial proportions of this range had low probability of exceeding 2% prevalence (figure 2C-D), particularly areas of higher uncertainty attributable to lower density sampling (Supplementary Material 2, Figure S4B). Moderate prevalence foci (≥ 10%) were identified in the north for both species, and in the east for *S. mansoni.* The most northern (Mashonaland Central) and southeastern (Masvingo) foci *for S. mansoni* represent the areas exhibiting greatest recrudescence in prevalence since 2018 (Supplementary Material 2, Table S1). Small, predicted foci for *S. haematobium* in the south of the country, not supported by empirical data (Figs. 1A and 2A), are attributable to the influence of survey inclusion probability (i.e. areas that were previously higher prevalence, but that were not sampled during this survey). Supplementary Material 2, Figure S7 delineates the contribution of survey inclusion probability in these areas. Figure 2E-F show the probability that each ward exceeds 10% prevalence, accounting for population-weighted estimates at 1 km resolution, highlighting sub-IUs where interventions are mostly likely needed using MoHCC treatment decision guidelines (Supplementary Material 2; Table S7).

**Fig. 3:**
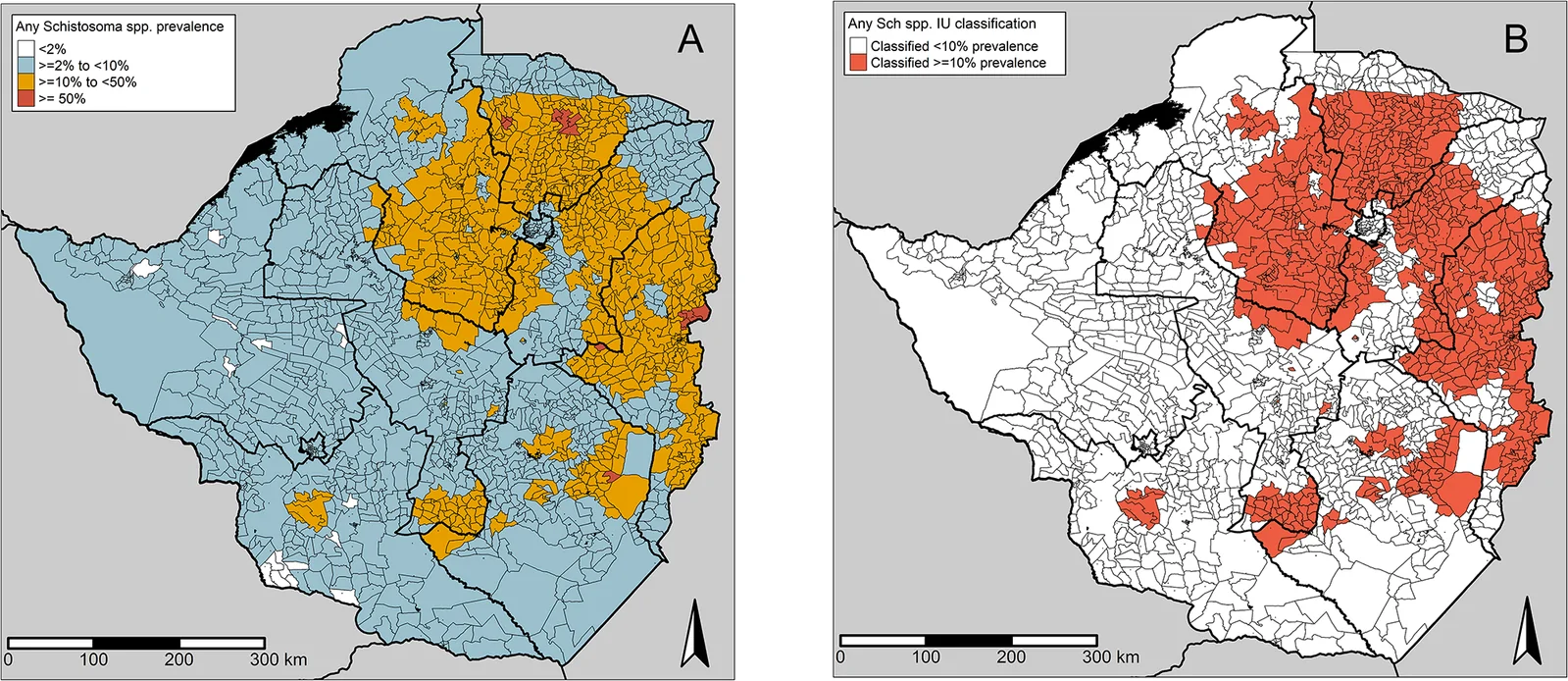
Ward level classification of infection with any schistosomiasis species determined by population weighted exceedance probability (**A**). Wards for which the probability of exceeding 10% prevalence was > = 30% (red) and indicated need for ongoing PC (**B**)

**Fig. 4:**
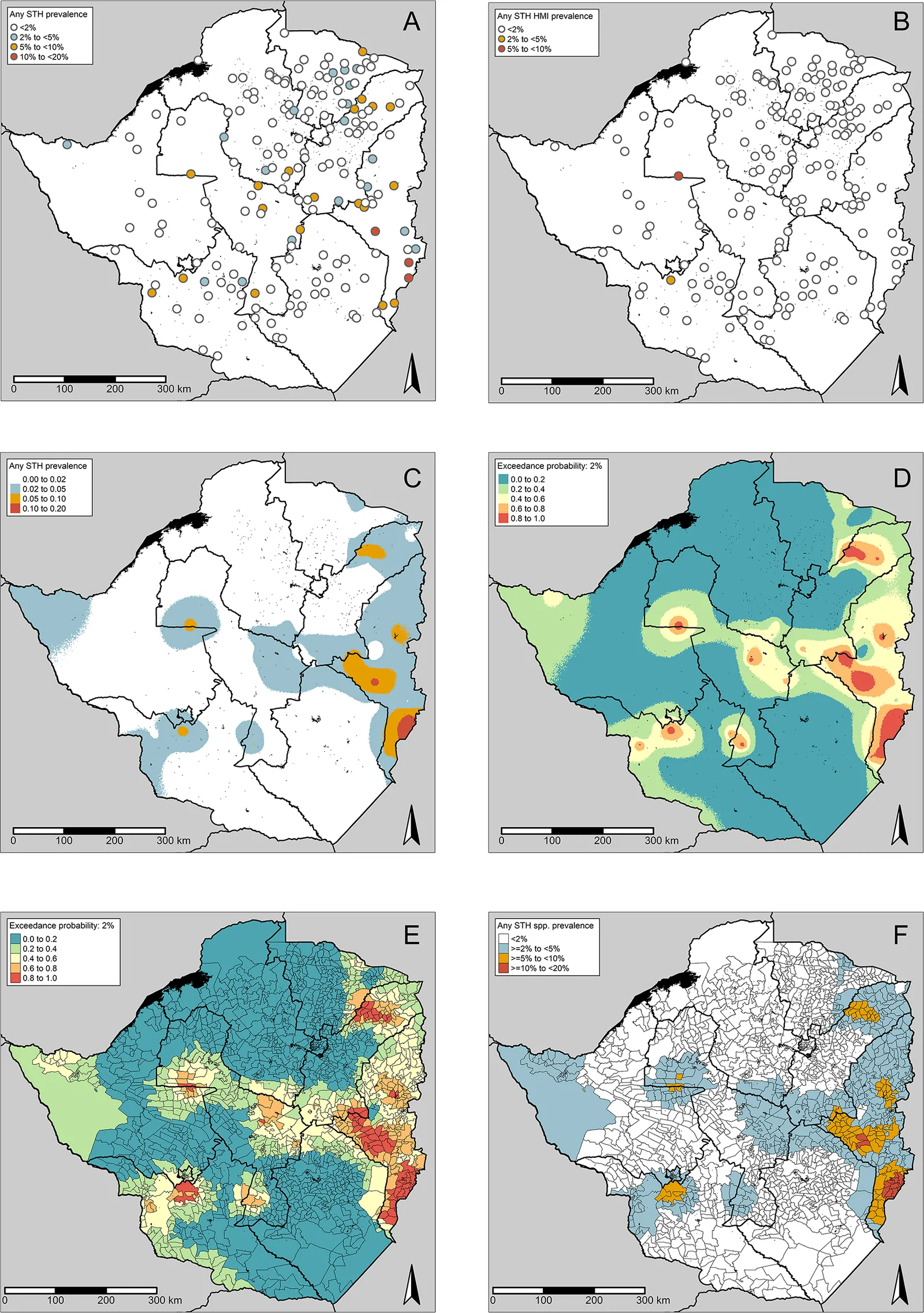
Site-level prevalence of infection (**A**) or, moderate or heavy intensity infection (**B**) with any STH species. Predicted mean prevalence of any STH infection (**C**). Estimated probability of exceeding 2% infection prevalence (**D**). Population weighted probability of exceeding 2% prevalence at ward-level for any STH infection (**E**) and final prevalence category classification (**F**)

All wards were classified into MoHCC prevalence categories for any schistosomiasis species, weighted by population density (Fig. 3A). These classifications informed PC intervention and surveillance strategies (Supplementary Material 2: Table S7), and we include total ward counts and estimated resident populations per category (Table 2). Nearly all wards (1940/1961, 98.9%) were considered > = 2% prevalence – that is, there were few (21/1961) wards where certainty of being below the 2% threshold exceeded 70%. Among wards classified as exceeding 2%, 730 wards (37.2%) exceeded 10% prevalence and require ongoing annual PC, and 16 wards (0.8%) were above 50% and may require intensified interventions. Figure 4B highlights all wards requiring ongoing annual PC, resident to an estimated 6.64 million people (36.9% of the total population; Table 2). From an implementation perspective, these wards form a contiguous area across the north and east, consistent with predicted distributions. Isolated higher-prevalence foci in the south warrant further investigation due to uncertainty in model predictions for these areas.

**Table 2:** Final classifications of wards and resident populations within MOHCC prevalence categories determined by geostatistical estimates for infection with any schistosomiasis (*Schistosoma spp*.) and soil-transmitted helminth (STH) species

Prevalence category	Total wards	Total population
**Any Schistosoma spp. infection**		
< 2%	21 (1.1%)	68,574 (0.4%)
>=2% to < 10%	1210 (61.7%)	11,268,916 (62.7%)
>=10% to < 50%	714 (36.4%)	6,473,455 (36.0%)
>= 50%	16 (0.8%)	167,061 (0.9%)
**Any STH infection**		
< 2%	1308 (66.7%)	12,244,321 (67.9%)
>=2% to < 5%	534 (27.2%)	4,636,100 (25.7%)
>=5% to < 10%	98 (5.0%)	924,253 (5.1%)
>= 10% to < 20%	21 (1.1%)	221,231 (1.2%)

### Soil transmitted helminthiases

The estimated national prevalence for infection with any STH species was 1.4% (95% CI 1.0-2.1%; Table 3). Nearly all infections (135/163, 82.8%) were caused by *A. lumbricoides* (1.2% national prevalence, 95% CI 0.8–1.9%) with limited occurrence of hookworm (0.1% 95%CI 0.1–0.2%) or *T. trichiura* (0.2% 95% CI 0.1–0.3%). Our updated estimates of STH indicate an almost doubling of national prevalence since 2018’s impact assessment (any STH infection; 0.7%, 95% CI 0.6–0.9%). Both *A. lumbricoides* and hookworm occurred in all eight sampled provinces, though at very low prevalence for hookworm, whilst *T. trichiura* was not detected in four provinces. Approximately two thirds of sites were absent of infection from any STH species (115/177, 64.9%; Fig. 4A). Only three sites remained over 10% prevalence (4.8%) and a limited proportion exhibited prevalence between 2 to < 5% (18, 10.2%) or 5 to < 10% (19, 10.7%). Provincial estimates of prevalence are presented in Table 3. These highlight STH occurrence in all eight provinces containing empirical data, with highest prevalence in Manicaland and Midlands. There were notable increases in prevalence from 2018 in Manicaland (2018 estimate 0.9% 95% CI 0.5–1.3%) and Mashonaland East (2018 estimate 1.0% 95% CI 0.4–1.5%) whilst other provinces remained largely stable. Importantly, we did not sample in Bulawayo which exhibited highest STH prevalence, though of highest uncertainty (2018 estimate 4.3% 95% CI 1.6–7.1%).

**Table 3:** Sample numbers and disaggregation by sex and age for each soil-transmitted helminth (STH) species (*Ascaris lumbricoides*, hookworm and *Trichuris trichiura*) outcome measure

	A. lumbricoides	Hookworm	T. trichiura	Any STH	Any STH high or moderate
Survey sites	177				
Examined (N)	10,676				
Infected (N)	135	18	14	153	5
**National prevalence (95% CI)**					
Overall	1.2 (0.8–1.9)	0.1 (0.1–0.2)	0.2 (0.1–0.3)	1.4 (1.0–2.1)	0.2 (< 0.1–1.2)
**Province prevalence (95% CI)**					
Manicaland	4.7 (2.9–7.4)	0.4 (0.1–1.1)	0.2 (< 0.1–0.7)	5.2 (3.2–8.1)	0 (0 - <0.1)
Mashonaland Central	0.5 (0.1–2.6)	0.3 (0.1–0.8)	0.1 (< 0.1–0.3)	0.7 (0.2–2.3)	0 (0 - <0.1)
Mashonaland East	2.1 (1.4–3.2)	0.1 (< 0.1–0.8)	0 (0 – <0.1)	2.2 (1.4–3.3)	0 (0 - <0.1)
Mashonaland West	0.2 (0.1–0.7)	< 0.1 (< 0.1–0.2)	0 (0 – <0.1)	0.2 (< 0.1–0.7)	0 (0 - <0.1)
Masvingo	0.2 (< 0.1–0.7)	0.1 (< 0.1–0.7)	0 (0 – <0.1)	0.2 (< 0.1–0.7)	0 (0 - <0.1)
Matabeleland North	0.3 (< 0.1–2.5)	0.2 (< 0.1–1.5)	0 (0 – <0.1)	0.5 (0.1–2.2)	0 (0 - <0.1)
Matabeleland South	0.9 (0.3–2.8)	< 0.1 (< 0.1–0.3)	0.9 (0.4–1.7)	1.8 (1.0–3.0)	< 0.1 (< 0.1–0.3)
Midlands	2.9 (0.9–9.0)	< 0.1 (< 0.1–0.3)	0.1 (< 0.1–1.0)	3.1 (1.0–8.9)	1.7 (0.3 - 8.8)

Design-based estimates of prevalence at national and province level

We estimated that the prevalence of heavy or moderate intensity infection with any STH species - and the associated upper confidence bound - was considerably below the STH EPHP threshold of 2% (0.2% 95% CI < 0.1–1.2%; Table 3). In absolute terms, we observed only 5 infections of moderate intensity infection, with four caused by *A. lumbricoides* and one by *T. trichiura* and no heavy intensity STH infections. Table 3 presents design-based estimates at province level, showing moderate intensity infections occurring within Midlands (*A. lumbricoides)* and Matabeleland (*T. trichiura*) at just two survey sites (Fig. 4B). Prevalence of moderate and heavy intensity infections had marginally increased from 0.1% to 0.2% by 2021 though with considerable uncertainty, suggesting little detectable change (Table 3).

Given the predominance of *A. lumbricoides* and limited distribution of other STH species, a single geostatistical model was fitted for infection with any STH species (Supplementary Material 2, Table S6, Figure S8). The final model did not include any environmental covariates, and the estimated range of spatial autocorrelation was larger than for schistosomiasis (practical range 67.3 km 95% CI 35.6-127.5; Supplementary Material, Table S7). Mean predicted prevalence maps (Fig. 4C) indicated that most of Zimbabwe was below 2% prevalence, with a band of 2 to < 10% prevalence along the eastern border and two distinct foci exceeding 5 and 10% prevalence. Notably, the two eastern foci within Matabeleland North were driven by single data points. Exceedance probability maps for > 2% prevalence at pixel and ward level (Fig. 4D-E), identify priority areas where transmission likely remains above the threshold. Population weighted estimates of ward level prevalence were then categorised by MoHCC definitions based on exceedance probability for intervention planning (Fig. 4E-F, Supplementary Material 2, Table S7) The updated categories (Table 2) indicated that 33.3% of wards and 5,781,584 (32.1%) of the population required ongoing biennial PC, though only 6.1% of these areas and 1,145,484 people (6.4%) required treatment of adults alongside SAC ( > = 5% prevalence).

## Discussion

This national reassessment confirms that Zimbabwe has broadly sustained its control gains for STH since the cessation of MDA in 2018 though not for schistosomiasis. Application of geostatistical methods enabled ward-level estimation of current burden nationwide and will facilitate precise targeting of future PC activities to address recrudescence. Of particular concern, mean prevalence of heavy intensity infection highly likely exceeded the 1% EPHP threshold nationally and within five provinces, ranging from 1.1% to 2.8%. This included areas where overall prevalence remained below 10%. Infection with either *Schistosoma* species increased generally across all endemic provinces, with *S. mansoni* showing evidence of particularly pronounced increases in Mashonaland Central and Masvingo. Masvingo province was the most striking given observed infection prevalence < 2% in 2018. There was also evidence of STH prevalence increasing, driven primarily by *A. lumbricoides* across two provinces, though consistently low and below PC intervention thresholds. These findings reaffirm the critical importance of continued surveillance following EPHP achievement and raise important questions about optimal PC frequency in settings with low but persistent transmission.

The suspension of PC between 2018 and 2021 was driven by external operational constraints, and the advent of COVID-19 which forced countries to impose Public Health and Social Measures including closure of schools (2020) and prevention of public gathering [15], rather than a deliberate policy shift by the MoHCC. Nevertheless, the interruption provides a valuable quasi-experimental case study for examining the implications of reduced PC intensity in previously treated populations. It has become of special relevance now, given that recent major cuts to global health funding have left NTD programmes severely under resourced, potentially delaying or stopping mass drug administration cycles [16]. While WHO guidelines currently permit reduced PC frequency when schistosomiasis and STH prevalence falls below 10%, there is limited empirical evidence to support these thresholds [5]. Recent transmission models have shown that in moderate to high transmission settings, scaling back PC after reaching EPHP can result in recrudescence, especially when scale-back occurs prematurely [17, 18]. These projections are consistent with field evidence from Zanzibar and Kenya, where schistosomiasis resurgence was observed following interruption of PC [19–21]. In Zimbabwe, most provinces in which we observed increased prevalence of heavy intensity infection were close to or above 10% prevalence in 2018. Further, a baseline survey in 2010 [9] prior to initiation of PC demonstrated high transmission potential in each (> 20% prevalence of infection with any *Schistosoma* species, with Mashonaland Central and Masvingo > 40%). These data also reinforce our interpretation that interruption of PC was the primary driver of the observed increase in prevalence, given that this rise contrasts with the declining trend during the PC implementation period (2010–2018). It is also important to note that effective rounds of PC were conducted in 7 districts during 2020, which likely mitigated the impact of PC interruption in these areas. In contrast, model-based studies for STH suggest that PC may be safely withdrawn at very low prevalence levels, provided that access to sanitation and hygiene is high; however, empirical evidence to support this approach remains limited [22, 23].

This study demonstrates the operational utility of model-based geostatistical frameworks for national NTD surveillance and decision-making [24, 25]. By incorporating prior prevalence data, environmental covariates, and spatial correlation structures, the MBG approach enabled optimisation of survey design [11], increased the resolution of prevalence estimates, and provided probabilistic assessments of whether key epidemiological thresholds (e.g., 2% or 10%) were exceeded at implementation unit level. This allowed the MoHCC to classify wards into appropriate treatment categories based on exceedance probabilities, rather than point prevalence alone. Notably, these gains in resolution and analytical power were achieved without increasing survey size, demonstrating the scalability and cost-effectiveness of this method for other endemic countries transitioning from district-level to community-level programme planning under the WHO 2030 NTD roadmap [26].

This study has several important limitations. First, while the geostatistical design improved sampling efficiency by targeting areas with higher predicted prevalence, some regions had sparse data, reducing estimate precision. In the south of Midlands and west of Matabeleland provinces for example, models predicted high *S. haematobium* prevalence above treatment thresholds - largely driven by included model covariates - despite few cases being detected, reflecting sampling limitations in low prevalence settings. Geostatistical modelling does not resolve this uncertainty but makes it visible, supporting risk-informed decisions such as conservative classification or targeted resampling to verify apparent absence. Second, environmental covariates were derived from publicly available global datasets, which may not fully capture local drivers of transmission such as detailed water contact behaviours, sanitation practices, or ecological factors influencing snail populations and parasite transmission, potentially limiting model precision. Third, adults were only sampled at select sites, limiting generalizability beyond school-aged children who were the main survey population. Finally, the survey was conducted over a two-month period in late 2021 and thus does not account for possible seasonal variation in infection prevalence.

## Conclusions

Zimbabwe’s 2021 reassessment illustrates the practical value of model-based geostatistical approaches for precision mapping and sub-district level targeting of schistosomiasis and STH interventions. These approaches supported more efficient, data-driven planning and can be replicated as countries progress to EPHP, in line with WHO’s 2030 roadmap. However, early signs of recrudescence point to the limitations of PC alone and they reinforce the need for adaptive surveillance systems capable of detecting and responding to changing transmission dynamics. To sustain progress, strengthened community engagement, health education and infrastructure investments are required [27, 28]. Integrating NTD surveillance data with broader health information systems has the potential to improve early detection and facilitate coordinated responses. In the long term, maintaining progress against NTDs will require not only better data and tools, but also strong, more integrated systems capable of sustaining impact over time.

## Supplementary Information

Below is the link to the electronic supplementary material.

Supplementary Material 1: Strobe Checklist.

Supplementary Material 2: Supplementary Methods and Results.

Supplementary Material 3: S. haematobium site-level prevalence data and extracted covariates.

Supplementary Material 4: S. mansoni site-level prevalence data and extracted covariates.

Supplementary Material 5: Covariates extracted across 1 km resolution grid extent of Zimbabwe.

Supplementary Material 6: Ward shapefile boundaries for Zimbabwe.

Supplementary Material 7: Analysis code in R.

Supplementary Material 8: Supporting R functions for analysis code.

## Data Availability

The datasets and code used and/or analysed during the current study are available within the manuscript and its supporting files.
