# Supplementary material for: A national geostatistical survey for schistosomiasis and soil-transmitted helminths in Zimbabwe following interruption of preventive chemotherapy
Source: BMC Glob Public Health. 2026 Sep 22;4:92. doi: 10.1186/s44263-026-00301-x (PMC13595540; doi:10.1186/s44263-026-00301-x)
Supplement: Supplementary file 1 [file 44263_2026_301_MOESM1_ESM.docx]

STROBE Statement—checklist of items that should be included in reports of observational studies

|  | Item No. | Recommendation | Page  No. | Relevant text from manuscript |
| --- | --- | --- | --- | --- |
| **Title and abstract** | 1 | (*a*) Indicate the study’s design with a commonly used term in the title or the abstract | 1 | A national geostatistical survey |
|  |  | (*b*) Provide in the abstract an informative and balanced summary of what was done and what was found | 1 | The cross-sectional survey in 2021 targeted 11,600 school-aged children (SAC) and sub-sets of pre-SAC and adults  Geostatistical models incorporating environmental covariates were fitted and used to generate high-resolution prevalence predictions.  PC suspension led to recrudescence of schistosomiasis and smaller increases in STH prevalence. |
| Introduction | | | |  |
| Background/rationale | 2 | Explain the scientific background and rationale for the investigation being reported | 3-4 | Recent guidance has also recommended transitioning schistosomiasis control from district- to community-level PC implementation (6). As a result, NTD programmes must now generate granular prevalence estimates to inform future PC strategies – prompting a shift in implementation units (IU) from district to sub-districts.  From 2018, schistosomiasis and STH control activities in Zimbabwe were disrupted for three years due to the Sars-Cov-2 pandemic and subsequent vaccination campaigns.  In response, the MoHCC prioritised a second national impact assessment survey, aligned with updated WHO guidance emphasising epidemiological data at finer spatial scales, specifically sub-district (ward) level. |
| Objectives | 3 | State specific objectives, including any prespecified hypotheses | 4 | Building on the 2018 geostatistical analysis (10), the 2021 national impact assessment adopted a model-based geostatistical (MBG) framework for both design and analysis. By leveraging spatial autocorrelation and associations with environmental covariates, this approach improves precision and allows reliable prediction in unsampled areas (11). The MBG approach also integrated prior data and uncertainty to optimise design and inference (12). Here, we present results from Zimbabwe’s 2021 impact assessment to update ward-level prevalence estimates to guide national decisions for schistosomiasis and STH. |
| Methods | | | |  |
| Study design | 4 | Present key elements of study design early in the paper | 1,4 | We conducted a nationwide geostatistical impact assessment to estimate prevalence at ward-level and evaluate impact of PC interruption to 2018 results using a quasi-experimental design.  A population-based cross-sectional survey was conducted between November and December 2021 targeting 11,600 school-aged children (ages 9-14 years) nationwide. We employed a geostatistical design and analysis framework in which schools were the primary sampling units (PSUs). Our primary aim was to correctly classify 90% wards (sub-districts) into prevalence categories defined by the MoHCC and WHO aligned for infection with any schistosomiasis species i) <2%, ii) 2 to <10%, iii) 10 to <50% and iv) ≥50%, and any STH species i) <2%, ii) 2 to <5%, iii) 5 to <10% and iv) ≥10%. We compared findings to those of a published 2018 nationwide impact survey as a quasi-experimental before-after study to quantify the impact of PC interruption. |
| Setting | 5 | Describe the setting, locations, and relevant dates, including periods of recruitment, exposure, follow-up, and data collection | 5 | A population-based cross-sectional survey was conducted between November and December 2021 targeting 11,600 school-aged children (ages 9-14 years) nationwide. |
| Participants | 6 | (*a*) *Cohort study*—Give the eligibility criteria, and the sources and methods of selection of participants. Describe methods of follow-up  *Case-control study*—Give the eligibility criteria, and the sources and methods of case ascertainment and control selection. Give the rationale for the choice of cases and controls  *Cross-sectional study*—Give the eligibility criteria, and the sources and methods of selection of participants | 5 | A population-based cross-sectional survey was conducted between November and December 2021 targeting 11,600 school-aged children (ages 9-14 years) nationwide.  The optimal design targeted 200 PSUs using a spatially-regulated design with selection proportional to exceedance probability of 2% prevalence for *S. haematobium*. Within PSUs, 58 children aged 9-14 and stratified by sex were selected per school. In a random sub-sample of locations (n = 25), pre-SAC and adults were sampled alongside SAC. |
|  |  | (*b*) *Cohort study*—For matched studies, give matching criteria and number of exposed and unexposed  *Case-control study*—For matched studies, give matching criteria and the number of controls per case |  |  |
| Variables | 7 | Clearly define all outcomes, exposures, predictors, potential confounders, and effect modifiers. Give diagnostic criteria, if applicable | 4-7  Supplementary Material 1: Table S3 | All study participants were asked to provide their name, age, sex, school grade and responses to a short set of behaviour-exposure questions, plus a sample of mid-morning urine and fresh stool. Urine and stool samples were examined on the day of collection. *Schistosoma haematobium* infection was assessed by urine filtration, with intensity of infection expressed as eggs per 10ml. *Schistosoma mansoni* and STH were assessed by duplicate Kato-Katz thick smears and intensity expressed as eggs per gram of feces.  Supplementary Material 1: Table S3  For each species, we first fitted a model without covariates. However, for each schistosomiasis species, inclusion probability was included as a fixed effect to account for the sampling design. We then added covariates selected from a suite of 30 across five risk domains (Table S3). Domains were defined based on the requirement that each provides an independent contribution to risk of schistosomiasis infection at the scales measured in the data. We did not limit the number of covariates selected per domain. |
| Data sources/ measurement | 8* | For each variable of interest, give sources of data and details of methods of assessment (measurement). Describe comparability of assessment methods if there is more than one group | Supplementary Material: Table S3 | Supplementary Material 1: Table S3 |
| Bias | 9 | Describe any efforts to address potential sources of bias | 20 | Not addressed directly given the geostatistical design and selection. General limitations and design outlined in the discussion:  First, while the geostatistical design improved sampling efficiency by targeting areas with higher predicted prevalence, some regions had sparse data, reducing estimate precision. In the south of Midlands and west of Matabeleland provinces for example, models predicted high *S. haematobium* prevalence above treatment thresholds - largely driven by included model covariates - despite few cases being detected, reflecting sampling limitations in low prevalence settings. Geostatistical modelling does not resolve this uncertainty but makes it visible, supporting risk-informed decisions such as conservative classification or targeted resampling to verify apparent absence. Second, environmental covariates were derived from publicly available global datasets, which may not fully capture local drivers of transmission such as detailed water contact behaviours, sanitation practices, or ecological factors influencing snail populations and parasite transmission, potentially limiting model precision. Third, adults were only sampled at select sites, limiting generalizability beyond school-aged children who were the main survey population. Finally, the survey was conducted over a two-month period in late 2021 and thus does not account for possible seasonal variation in infection prevalence. |
| Study size | 10 | Explain how the study size was arrived at | 5 | We then simulated potential survey designs across predicted realizations - varying sampling designs, analysis approaches, sites, and PSUs sampled – to specify a design which correctly classified 90% of wards with minimal survey effort (Figure S2-3). The optimal design targeted 200 PSUs using a spatially-regulated design with selection proportional to exceedance probability of 2% prevalence for *S. haematobium*. |

Continued on next page

| Quantitative variables | 11 | Explain how quantitative variables were handled in the analyses. If applicable, describe which groupings were chosen and why | 7 | For each species, we first fitted a model without covariates. However, for each schistosomiasis species, inclusion probability was included as a fixed effect to account for the sampling design. We then added covariates selected from a suite of 30 across five risk domains (Table S3). Domains were defined based on the requirement that each provides an independent contribution to risk of schistosomiasis infection at the scales measured in the data. We did not limit the number of covariates selected per domain. All covariates were screened for collinearity and removed if pairwise Pearson correlation was ≥ 0.8, then added in a stepwise fashion in a non-spatial framework. Each mode at this stage was evaluated for improvement in model fit to determine inclusion of a covariate in a final geostatistical model. |
| --- | --- | --- | --- | --- |
| Statistical methods | 12 | (*a*) Describe all statistical methods, including those used to control for confounding | 6 | We calculated national and provincial prevalence estimates using design-based inference under a one-stage cluster design with PSUs weighted by the reciprocal of inclusion probability. We estimated robust confidence intervals using Wald’s method.  School-level prevalence data were then analysed using a geostatistical modelling framework, an extension of mixed-effects logistic regression that incorporates spatial autocorrelation between PSUs and supports interpolation of prevalence into unsampled areas. For each species, we fitted a geostatistical model as follows: |
|  |  | (*b*) Describe any methods used to examine subgroups and interactions |  | NA |
|  |  | (*c*) Explain how missing data were addressed | 8 | Complete case analysis used:  Complete datasets were available from 9,778 SAC in 177 schools across eight provinces (two urban provinces – Bulawayo and Harare – had no schools selected during sampling; Figure S4). |
|  |  | (*d*) *Cohort study*—If applicable, explain how loss to follow-up was addressed  *Case-control study*—If applicable, explain how matching of cases and controls was addressed  *Cross-sectional study*—If applicable, describe analytical methods taking account of sampling strategy | 6 | School-level prevalence data were then analysed using a geostatistical modelling framework, an extension of mixed-effects logistic regression that incorporates spatial autocorrelation between PSUs and supports interpolation of prevalence into unsampled areas. |
|  |  | (*e*) Describe any sensitivity analyses |  | NA |
| Results | | | | |
| Participants | 13* | (a) Report numbers of individuals at each stage of study—eg numbers potentially eligible, examined for eligibility, confirmed eligible, included in the study, completing follow-up, and analysed | 5,8 | The optimal design targeted 200 PSUs using a spatially-regulated design with selection proportional to exceedance probability of 2% prevalence for *S. haematobium*. Within PSUs, 58 children aged 9-14 and stratified by sex were selected per school.  Complete datasets were available from 9,778 SAC in 177 schools across eight provinces (two urban provinces – Bulawayo and Harare – had no schools selected during sampling; Figure S4). |
|  |  | (b) Give reasons for non-participation at each stage |  |  |
|  |  | (c) Consider use of a flow diagram |  | NA |
| Descriptive data | 14* | (a) Give characteristics of study participants (eg demographic, clinical, social) and information on exposures and potential confounders | 8 | Complete datasets were available from 9,778 SAC in 177 schools across eight provinces (two urban provinces – Bulawayo and Harare – had no schools selected during sampling; FFigure S4). In addition, data from 725 PSAC and 750 adults were also collected from the sub-sample of 25 sites. The sample population was split evenly by sex (Table 1) and the mean age of the sample population was 12.3 years (SD 7.4). The median distance between PSUs was 19.8 km (IQR 13.7 – 30.2; Figure S4). |
|  |  | (b) Indicate number of participants with missing data for each variable of interest |  | NA – complete data available for geostatistical analysis (earth observation data per pixel) |
|  |  | (c) *Cohort study*—Summarise follow-up time (eg, average and total amount) |  |  |
| Outcome data | 15* | *Cohort study*—Report numbers of outcome events or summary measures over time |  |  |
|  |  | *Case-control study—*Report numbers in each exposure category, or summary measures of exposure |  |  |
|  |  | *Cross-sectional study—*Report numbers of outcome events or summary measures | 9 | Table 1 |
| Main results | 16 | (*a*) Give unadjusted estimates and, if applicable, confounder-adjusted estimates and their precision (eg, 95% confidence interval). Make clear which confounders were adjusted for and why they were included |  | Survey-adjusted findings reported in Table 1 |
|  |  | (*b*) Report category boundaries when continuous variables were categorized |  | NA – not used |
|  |  | (*c*) If relevant, consider translating estimates of relative risk into absolute risk for a meaningful time period | 9-16 | Estimates and predictions provided on prevalence risk scale. |

Continued on next page

| Other analyses | 17 | Report other analyses done—eg analyses of subgroups and interactions, and sensitivity analyses |  | NA |
| --- | --- | --- | --- | --- |
| Discussion | | | | |
| Key results | 18 | Summarise key results with reference to study objectives | 18 | This national reassessment confirms that Zimbabwe has broadly sustained its control gains for STH since the cessation of MDA in 2018 though not for schistosomiasis. Application of geostatistical methods enabled ward-level estimation of current burden nationwide and will facilitate precise targeting of future PC activities to address recrudescence. |
| Limitations | 19 | Discuss limitations of the study, taking into account sources of potential bias or imprecision. Discuss both direction and magnitude of any potential bias | 20 | This study has several important limitations. First, while the geostatistical design improved sampling efficiency by targeting areas with higher predicted prevalence, some regions had sparse data, reducing estimate precision. In the south of Midlands and west of Matabeleland provinces for example, models predicted high *S. haematobium* prevalence above treatment thresholds - largely driven by included model covariates - despite few cases being detected, reflecting sampling limitations in low prevalence settings.  Geostatistical modelling does not resolve this uncertainty but makes it visible, supporting risk-informed decisions such as conservative classification or targeted resampling to verify apparent absence. Second, environmental covariates were derived from publicly available global datasets, which may not fully capture local drivers of transmission such as detailed water contact behaviours, sanitation practices, or ecological factors influencing snail populations and parasite transmission, potentially limiting model precision. Third, adults were only sampled at select sites, limiting generalizability beyond school-aged children who were the main survey population. Finally, the survey was conducted over a two-month period in late 2021 and thus does not account for possible seasonal variation in infection prevalence. |
| Interpretation | 20 | Give a cautious overall interpretation of results considering objectives, limitations, multiplicity of analyses, results from similar studies, and other relevant evidence | 20 | Zimbabwe’s 2021 reassessment illustrates the practical value of model-based geostatistical approaches for precision mapping and sub-district level targeting of schistosomiasis and STH interventions.  However, early signs of recrudescence point to the limitations of PC alone and they reinforce the need for adaptive surveillance systems capable of detecting and responding to changing transmission dynamics. To sustain progress, strengthened community engagement, health education and infrastructure investments are required (20,21). |
| Generalisability | 21 | Discuss the generalisability (external validity) of the study results | 20 | These approaches supported more efficient, data-driven planning and can be replicated as countries progress to EPHP, in line with WHO’s 2030 roadmap. |
| Other information | |  | | |
| Funding | 22 | Give the source of funding and the role of the funders for the present study and, if applicable, for the original study on which the present article is based | 21 | The study was funded through the END Fund’s Deworming Innovation Fund which receives funding from anchor partners including The ELMA Foundation, Delta Philanthropies, the Children’s Investment Fund Foundation, the Wyss Medical Foundation, and Virgin Unite through an Audacious Project. These source funding bodies had no role in study recruitment, data collection, data analysis, or data interpretation or involvement in draft or final manuscripts. |

*Give information separately for cases and controls in case-control studies and, if applicable, for exposed and unexposed groups in cohort and cross-sectional studies.

**Note:** An Explanation and Elaboration article discusses each checklist item and gives methodological background and published examples of transparent reporting. The STROBE checklist is best used in conjunction with this article (freely available on the Web sites of PLoS Medicine at http://www.plosmedicine.org/, Annals of Internal Medicine at http://www.annals.org/, and Epidemiology at http://www.epidem.com/). Information on the STROBE Initiative is available at www.strobe-statement.org.
