# Supplementary material for: A national geostatistical survey for schistosomiasis and soil-transmitted helminths in Zimbabwe following interruption of preventive chemotherapy
Source: BMC Glob Public Health. 2026 Sep 22;4:92. doi: 10.1186/s44263-026-00301-x (PMC13595540; doi:10.1186/s44263-026-00301-x)
Supplement: Supplementary file 2 [file 44263_2026_301_MOESM7_ESM.docx]

## **A national geostatistical survey for schistosomiasis and soil-transmitted helminths in Zimbabwe following interruption of preventive chemotherapy - Supplementary Material 1: Methods and Results**

### 2018 impact assessment results

These results are presented as reference values - at both national and province level – to estimate change in schistosomiasis and STH prevalence results following cessation of PC in 2019. The MoHCC conducted national impact assessment for schistosomiasis and STH between September-December 2018. Methods are included in Midzi et al 2020 (9) but in brief, schools were PSUs as part of a stratified one-stage cluster design to estimate prevalence at province and district level. A total of 336 PSUs were sampled proportional to size within district strata with 50 SAC aged 9-14 sampled per school.

*S. haematobium* was identified as the predominant schistosomiasis species with a mean prevalence of (3.0% 95% CI 2.7-3.3) with highest prevalence observed in all three Mashonaland provinces. The mean prevalence of *S. mansoni* was (2.4% 95% CI 2.2-2.7) and mainly in Manicaland provinces (table S1). The national prevalence of infection with any schistosomiasis was (5.1% 95% CI 4.7-5.5). For STH the predominant species was *A. lumbricoides* (0.5% 95% CI 0.4-0.7) than hookworm (0.2% 95% CI 0.1-0.3) or *T. trichiura* (0.1% 95% CI <0.1-0.1). For any STH the estimated prevalence was 0.7% (95% CI 0.6-0.9) and prevalence of moderate or heavy intensity infection was 0.1% (Midzi et al., 2020). Prevalence maps are presented in Midzi et al., 2020 that demonstrate two foci of STH infection >2%, in the northeast (Mashonaland Central) and the other towards the southern area of Midlands and surrounding province borders.

**Table S1:** 2018 impact assessment prevalence estimates for schistosomiasis in Zimbabwe. Estimates denoting design adjusted estimates of prevalence at national and province level.

|  | ***S. mansoni*** | **Heavy intensity** | ***S. haematobium*** | **Heavy intensity** | **Any *Schistosoma***  ***Spp.*** | **Heavy intensity** |
| --- | --- | --- | --- | --- | --- | --- |
| **National prevalence (95% CI)** | | | | |  |  |
| Overall | 2.4  (2.2-2.7) | 0  (0-<0.1) | 3.0  (2.7-3.3) | 0.4  (0.3-0.6) | 5.1  (4.7-5.5) | 0.4  (0.3-0.6) |
| **Province prevalence (95% CI)** | | | | |  |  |
| Manicaland | 8.8  (7.7 – 10.1) | 0  (0 – <0.1) | 2.8  (2.2 – 3.6) | 0.4  (0.2-0.8) | 10.5  (9.3-11.8) | 0.4  (0.2-0.8) |
| Mashonaland Central | 3.1  (2.4-4.1) | 0  (0 – <0.1) | 7.6  (6.4-9.0) | 0.8  (0.5-1.4) | 10.2  (8.8-11.8) | 0.8  (0.5-1.4) |
| Mashonaland East | 5.1  (4.1 – 6.4) | 0  (0– <0.1) | 3.8  (2.9-4.9) | 1.0  (0.6-1.6) | 8.1  (6.8-9.6) | 1.0  (0.6-1.6) |
| Mashonaland West | 0  (0- <0.1) | 0  (0 – <0.1) | 4.4  (3.5-5.5) | 0.5  (0.3-1.0) | 4.4  (3.5-5.5) | 0.5  (0.3-1.0) |
| Masvingo | 0.7  (0.4 - 1.5) | 0  (0 – <0.1) | 1.2  (0.8-1.5) | 0.3  (0.1-0.7) | 1.9  (1.4-2.6) | 0.3  (0.1-0.7) |
| Matabeleland North | 0  (0 - <0.1) | 0  (0 – <0.1) | 0.7  (0.3-1.5) | 0  (0 – <0.1) | 0.7  (0.3-1.5) | 0  (0 – <0.1) |
| Matabeleland South | 0  (0 – <0.1) | 0  (0 – <0.1) | 2.9  (2.1-4.1) | 0.3  (<0.1-0.9) | 2.9  (2.1-4.1) | 0.3  (0-0.9) |
| Midlands | 0.1  (<0.1– 0.5) | 0  (0 – <0.1) | 2.0  (1.5-2.8) | 0  (0 – <0.1) | 2.1  (1.5-2.9) | 0  (0 – <0.1) |

### Use of 2018 impact assessment results to inform 2021 geostatistical survey design

To design the 2021 reassessment survey, we used data from 2018 to design a sampling and analysis approach that would ensure >90% of IUs were correctly classified with minimal survey effort. We generated a geostatistical risk map for *S. haematobium*, given its status as the predominant schistosomiasis species in Zimbabwe. Models were fitted using the approach outlined in the main methods. We present the estimated model parameters in table S2, and estimated prevalence at 5km scale figure S1A. Across 1000 geostatistical realisations, we simulated potential survey designs and estimated the prevalence of every ward in Zimbabwe from the results of each simulation. Estimated ward prevalence was then compared with the true classification per realisation. We varied the following parameters to identify a survey design which achieved 90% correct classification of wards for minimum sampling effort:

1. Number of PSUs to sample (proportion of schools within the original sample)
2. Number of SAC sampled per PSU
3. Locations of PSU sampled: randomly sampled, spatially regulated, spatially regulated plus close pairs
4. Use of inclusion probability for PSUs defined by their probability of exceeding 2% prevalence.

Results of simulations shown in figure S2-3. The optimal approach used a spatial coverage sample with the probability of selection additionally defined by exceedance probability of 2% prevalence. Figure S1B shows the predicted exceedance probability of 2% at 5km scale from the fitted model of *S. haematobium* used to define inclusion probabilities.

**Table S2:** Parameters estimates for final geostatistical model for *S. haematobium* using 2018 impact assessment data*.* Covariates were scaled and centred prior to model fitting and presented on log odds scale.

| **Parameter** | **Estimate** | **95% CI** |
| --- | --- | --- |
| Intercept | -7.38 | (-13.65, -1.11) |
| Distance to nighttime light | -0.189 | (-4.01, 0.02) |
| NDVI mean | 3.43 | (-0.28, 7.15) |
| Soil pH | -0.01 | (-0.01, 0) |
| Population | 0.07 | (-0.05, 0.19) |
| Distance to rivers | -0.02 | (-0.05, 0.01) |
| Soil moisture | 0.01 | (-0.01, 0.04) |
| Sigma^2 | 1.40 | (0.85, 2.31) |
| Phi | 34.82 | (18.33, 66.15) |
| Tau^2 | 0.87 | (0.21. 3.56) |
| Kappa | 0.5 | - |

**Figure S1:** Estimated mean prevalence of infection with S. haematobium in Zimbabwe from geostatistical models fitted to 2018 impact assessment data (A). Estimated probability of exceeding 2% prevalence (B) from the same model. Location of sampled school with empirical *S. haematobium* infection prevalence from 2018 survey (C). Histogram of empirical school-level prevalence distribution from 2018 (D).

**A**

**B**

**A**

**C**

**D**

**Figure S2:** Results of survey designs simulated across fitted realisations of *S. haematobium* prevalence from 2018 impact assessment data. Results shown varied total sites sampled (x-axis), SAC sampled per site, analysis method (green; MBG (model-based geostatistics)) and site selection approaches; i) random ii) regular spatial grid iii) regular spatial grid plus 10% close pairs iv) regular spatial grid plus 30% close pairs.

**Figure S3:** Results of additional survey designs simulated across fitted realisations of *S. haematobium* prevalence from 2018 impact assessment data. Results shown varied total sites sampled (x-axis), analysis method (green; MBG) and site selection approaches; i) random ii) regular spatial grid. Relative to figure S3, these approaches all included the selection of sites by probability proportional to exceedance probability of 2% prevalence of *S. haematobium*.

**Table S3:** Covariates included for exploration in geostatistical models

| **Variable** | **Resolution** | **Source** |
| --- | --- | --- |
| **Sociodemographic and health care access** | | |
| WorldPop Population density - Unconstrained 2020 UN adjusted | 1km | <https://hub.worldpop.org/geodata/summary?id=46094> |
| Gross domestic product (PPP) | 1000m | <https://datadryad.org/stash/dataset/doi:10.5061/dryad.dk1j0> |
| Nighttime light intensity (VIIRS): Distance to stable night lights | 500m | <https://eogdata.mines.edu/products/vnl/> |
| Malaria Atlas Project motorized time to health facility (Friction surface) | 1000m | <https://malariaatlas.org/research-project/accessibility-to-healthcare/> |
| IHME measles vaccine coverage (First dose 2019) | 5000m | <https://ghdx.healthdata.org/record/ihme-data/lmic-mcv1-coverage-geospatial-estimates-2000-2019> |
| **Bioclimatic** | | |
| GMTED elevation data | 90m | [https://earthexplorer.usgs.gov](https://earthexplorer.usgs.gov/) |
| WorldClim variables: |  |  |
| - Long-term temperature (1970-2000): mean; seasonality; range | 1km | <https://www.worldclim.org/data/bioclim.html> |
| - Long-term precipitation (1970-2000): annual mean; precipitation of driest month; seasonality | 1km | <https://www.worldclim.org/data/bioclim.html> |
| **Landcover** | | |
| NDVI 2015-2019 (mean, sd and minimum) | 100m | <https://land.copernicus.eu/global/products/ndvi> |
| opernicus Land Monitoring Service classification variables - distance to land covered by: | | |
| - bare soil; crops; grassland; shrubs; | 100m | [https://zenodo.org/record/3939050#.Yjm7Ti2l29Z](https://zenodo.org/record/3939050) |
| **Geological** | | |
| - Soil pH | 1000m | <https://envirometrix.nl/isdasoil-open-soil-data-for-africa/> |
| - Soil water index (static) | 12500m | <https://www.copernicus.eu/en/access-data/copernicus-services-catalogue/soil-water-index-static-layers-raster-125-km-global> |
| - Flow accumulation | 1000m | Manually derived from GMTED elevation data |
| **Exposure to contaminated water** | | |
| Global surface water estimated distance to: | | |
| - any water body occurrence in 2021 | 100m | <https://global-surface-water.appspot.com/download> |
| - permanent water body 2021 | 100m |  |
| - seasonal water body (3-9 months coverage) in 2021 | 100m |  |
| - any river | 90m | <https://www.hydrosheds.org/products/hydrolakes> |
| IHME water, sanitation and hygiene indices: | | |
| - reliance upon surface water | 5000m | <https://ghdx.healthdata.org/record/ihme-data/lmic-wash-access-geospatial-estimates-2000-2017> |
| - to improved water | 5000m |  |
| - access to improved sanitation | 5000m |  |
| - reliance upon open defecation | 5000m |  |

### Province names and distance between PSUs

Figure S4A maps the names of provinces to which design-based estimates were aggregated to, as a spatial reference for those less familiar with the geography of Zimbabwe. Figure S4B shows the distance of every 1km pixel in Zimbabwe from its closest survey point (PSU). This figure in conjunction with tables S4-5 indicate areas where model estimates should be interpreted cautiously.

**Figure S4:** Names of provinces in Zimbabwe overlaid within their administrative boundaries (A). Distance of all 1km pixels in Zimbabwe to their nearest survey location (in metres).

**B**

**A**

### Parameter estimates of geostatistical models

We present the final model estimates for all parameters in geostatistical models for *S. haematobium* (table S4) and *S. mansoni* (table S5) as well as the validation variograms for each fitted model (figure S5-6). Figure S7 shows the separate predictions across Zimbabwe for the linear predictor component of the model (A) and the spatial Gaussian process (B). We present these to highlight the strong influence of the linear predictor for *S. haematobium* that was mainly driven by survey inclusion probability (probability of exceeding 2% prevalence) and contributes to some discordant observations between empirical and predicted prevalence in south Zimbabwe.

**Table S4:** Parameters estimates for final geostatistical model for *S. haematobium* using 2021 reassessment data*.* Covariates were scaled and centred prior to model fitting and presented on log odds scale.

| **Parameter** | **Estimate** | **95% CI** |
| --- | --- | --- |
| Intercept | -3.67 | (-4.00, -3.35) |
| Exceedance probability of 2% prevalence (2018 estimate) | 1.10 | (0.78, 1.42) |
| Sigma^2 | 2.61 | (1.95, 3.48) |
| Phi | 8.17 | (2.68, 24.88) |
| Tau^2 | 0 | - |
| Kappa | 0.5 | - |

**Figure S5:** Residuals of observed *S. haematobium* model, where the spatial Gaussian process is set to zero, within an envelope (grey shaded area) derived from 1,000 semivariograms simulated from the full fitted model.-

**Table S5:** Parameters estimates for final geostatistical model for *S. mansoni* using 2021 reassessment data*.* Covariates were scaled and centred prior to model fitting and presented on log odds scale.

| **Parameter** | **Estimate** | **95% CI** |
| --- | --- | --- |
| Intercept | -4.53 | (-4.89, -4.17) |
| Exceedance probability of 2% prevalence (2018 estimate) | 0.70 | (0.334, 1.07) |
| Precipitation: Annual mean | 0.05 | (-0.42. 0.52) |
| Temperature: Annual range | -0.227 | (-0.67, 0.21) |
| Proportion of landcover as grassland | 0.13 | (-0.13, 0.39) |
| Purchasing power parity | 0.46 | (0.20, 0.71) |
| 1^st^ dose Measles vaccination coverage | -0.41 | (-0.77, -0.06) |
| Sigma^2 | 2.72 | (2.14, 3.46) |
| Phi | 18.7 | (11.88, 29.39) |
| Tau^2 | 0 | - |
| Kappa | 0.5 | - |

**Figure S6:** Residuals of observed *S. mansoni* model, where the spatial Gaussian process is set to zero, within an envelope (grey shaded area) derived from 1,000 semivariograms simulated from the full fitted model.-

**Figure S7:** Mean estimates of the linear predictor (A) and spatial Gaussian process $S\left( x \right)_{i}$(B) for models fitted to *S. haematobium* using 2021 data.

**A**

**B**

**Table S6:** Parameters estimates for final geostatistical model for any STH species using 2021 reassessment data*.* Covariates were scaled and centred prior to model fitting and presented on log odds scale.

| **Parameter** | **Estimate** | **95% CI** |
| --- | --- | --- |
| Intercept | -5.11 | (-5.93, -4.28) |
| Sigma^2 | 2.63 | (1.49, 4.65) |
| Phi | 67.3 | (35.6, 127.46) |
| Tau^2 | 0 | - |
| Kappa | 0.5 | - |

**Figure S8**: Residuals of observed model for infection with any species of STH, where the spatial Gaussian process is set to zero, within an envelope (grey shaded area) derived from 1,000 semivariograms simulated from the full fitted model.-

### Programmatic use of geostatistical results

### The MOHCC in Zimbabwe utilised ward-level outputs to determine future treatment strategies. The following table denotes the future treatment strategy based on 2018 results and subsequent classification by geostatistical methods in 2021.

**Table S7:** Decision algorithm for future intervention strategy using ward-level classification results in conjunction with historical data (T-T denotes test and treat strategy).

| **Schistosomiasis** | | | | |
| --- | --- | --- | --- | --- |
| Baseline Results*  (prevalence) | Impact Results*  (prevalence) | Reassessment Results | WHO 2022 guidelines | MOHCC decision |
| **No** | <10% | <10% | T-T or maintain/reduce PC (SAC only) | T-T or maintain/reduce PC (SAC only) |
| **Low** | No | <10% | T-T or maintain/reduce PC (SAC only) | T-T (all age groups) |
| **Moderate** | No | <10% | T-T or maintain/reduce PC (SAC only) | T-T (all age groups) |
| **Moderate** | Low/Moderate | <10% | T-T or maintain/reduce (SAC only) | maintain/reduce (SAC only) |
| **Moderate** | Low/Moderate | >10% | annual PC (all age groups) | annual PC (all age groups) |
| **High** | Low/Moderate | >10% | annual PC (all age groups) | annual PC (all age groups) |
| **STH** | | | | |
| Baseline Results^#^  (prevalence) | Impact Results^#^  (prevalence) | Reassessment Results | WHO 2011 / 2017 guidelines | MOHCC decision |
| **Low** | No / <2% | <2% | No treatment | No treatment |
| **Low** | Low | <2% | No treatment | No treatment |
| **Moderate/High** | Low | <2% | No treatment | No treatment or T-T |
| **Low** | No / <2% | 2% - 5% | Biennial PC (SAC only) | Biennial PC (SAC only) |
| **Low** | Low | 2% - 5% | Biennial PC (SAC only) | Biennial PC (SAC only) |
| **Moderate/High** | Low/Moderate | 2% - 5% | Biennial PC (SAC only) | Biennial PC (SAC only) |
| **Low** | No / <2% | 5% - 10% | Biennial PC (SAC only) | Biennial PC (SAC & Adults (WRA)) |
| **Low** | Low | 5% - 10% | Biennial PC (SAC only) | Biennial PC (SAC & Adults (WRA)) |
| **Moderate/High** | Low/Moderate | 5% - 10% | Biennial PC (SAC only) | Biennial PC (SAC & Adults (WRA)) |
